# Childhood Maltreatment in Individuals With Schizophrenia Spectrum Disorders: The Impact of Cut-Off Scores on Prevalence Rates

**DOI:** 10.3389/fpsyt.2021.692492

**Published:** 2021-07-06

**Authors:** Angelina Weitkämper, Michael Kellner, Jona Ruben Iffland, Martin Driessen, Hanna Kley, Frank Neuner, Benjamin Iffland

**Affiliations:** ^1^Department of Clinical Psychology and Psychotherapy, Bielefeld University, Bielefeld, Germany; ^2^Department of Psychiatry, Psychotherapy and Psychosomatics, Hospital Herford, Herford, Germany; ^3^Department of Psychiatry and Psychotherapy, University Hospital Hamburg-Eppendorf, Hamburg, Germany; ^4^Centre for Psychiatry, Justus Liebig University Gießen, Gießen, Germany; ^5^Clinic of Psychiatry and Psychotherapy Bethel, Bielefeld, Germany; ^6^Department of Clinical Psychology and Psychotherapy, Outpatient Clinic, Bielefeld University, Bielefeld, Germany

**Keywords:** schizophrenia spectrum disorders, child neglect, child abuse, childhood maltreatment, peer victimization, prevalence of childhood maltreatment

## Abstract

Childhood maltreatment is a common phenomenon in various psychiatric disorders. Accordingly, patients with disorders from the schizophrenia spectrum (SSD) appear to have high prevalence rates of childhood maltreatment. However, the interpretation and comparability of prevalence rates is impeded by methodological weaknesses and differences such as measures and thresholds used in previous studies. Therefore, we aimed to provide and compare systematically captured data on prevalence rates of all common types of childhood maltreatment in patients with SSD using a standardized and well-established questionnaire and the most frequently used thresholds. The sample consisted of 48 patients with a primary diagnosis of SSD. 58.3–77.1% of the sample experienced at least one type of childhood maltreatment. Prevalence rates for physical abuse, physical neglect, and emotional abuse were dependent on the thresholds used, while equal rates were found for emotional neglect and sexual abuse. Physical neglect (46–67%), and emotional abuse (44–48%) were most commonly reported, followed by emotional neglect (38%), physical abuse (25–38%), and sexual abuse (25%). Additionally, high levels of peer victimization were reported by SSD patients. It appears that childhood maltreatment is a common phenomenon in SSD, even though methodological details, especially cut-off scores, have a substantial impact on the prevalence rates that are determined. Therefore, the methodology of studies should be closely examined when drawing conclusions from presented prevalence rates.

## Introduction

One of the most important challenges worldwide is childhood maltreatment (1). Childhood maltreatment refers to all forms of physical, emotional, or sexual abuse, neglect, or transgressions resulting in actual or potential harm to the child's health, survival, or development (2). Generally, experiences of childhood maltreatment appear to be an important risk factor for mental health problems (3) and are associated with various psychiatric disorders such as substance use disorder, depression, anxiety disorders, and eating disorders (3–5). Individuals who have experienced childhood maltreatment present with an earlier onset of symptomatology, a greater symptom severity, more hospitalizations and more suicide attempts (6–11).

A growing body of research has investigated childhood maltreatment in disorders of the schizophrenia spectrum (SSD), resulting in a change in the conceptualization of SSD leading from a rather narrow focus on genes and brain functions to an integrated bio-psycho-social approach (12). Accordingly, several studies reported that childhood maltreatment is also very common in SSD with prevalence rates ranging between 18 and 87% (8, 13–18). In line with findings in other psychiatric disorders, SSD patients who have experienced childhood maltreatment exhibited higher symptom severity, more co-morbid disorders, greater cognitive impairment, an earlier age at first hospitalization, more hospitalizations, and more suicide attempts than SSD patients without childhood maltreatment experiences (8, 10, 17). Thus, childhood maltreatment is not only a common phenomenon in SSD but also appears to be associated with a less favorable course of disease.

However, previous studies reported a wide range of rates of childhood maltreatment in cases of SSD (8, 14–17, 19–30). The wide range may be due to real differences in different samples but also to methodical differences. For instance, childhood maltreatment was assessed using different methods (e.g., interviews, questionnaires) and different measures [e.g., Childhood Trauma Questionnaire (CTQ; 2), Maltreatment and Abuse Chronology of Exposure – Scale (MACE; 34)].

For example, Kim et al. (21) used a semi-structured interview on the history of sexual and physical abuse, based on Russell's typology (31) of severe and very severe sexual abuse and the operationalization of physical abuse in the Conflict Tactics Scale (32). The authors reported prevalence rates of 19% for sexual abuse and 24.7% for physical abuse. Rubino et al. (27) reported a slightly higher prevalence rate of 28.7% of at least one type of childhood maltreatment while using a semi-structured interview [the Abuse Questionnaire; (33)]. However, the authors assessed physical and sexual abuse as well as emotional and psychological abuse which may explain the higher frequency of childhood maltreatment. Husted et al. (14) used a modified structured clinical interview for DSM-IV and additional data sources (medical, social and legal records and collateral information provided by other family members) to assess childhood maltreatment and reported a relatively low prevalence rate of 18.4%. Schenkel et al. (8) also used a detailed structured interview supplemented by information from the medical chart. The authors reported a prevalence rate of at least one type of childhood maltreatment more than twice as high (45.1%). Whereas, Husted et al. (14) defined childhood maltreatment “as a threat to physical, emotional and sexual integrity, a victim of serious injury or illness or a witness of violence” (p. 3) with no distinction of subtypes, Schenkel et al. (8) only captured sexual and physical abuse.

When questionnaires have been used to capture childhood maltreatment, prevalence rates tended to be higher compared to data collected via interviews. In two studies, sexual abuse alone was captured using a 4-item screening questionnaire, which was created for a Canadian survey, and prevalence rates of 35 and 40% were reported (22, 23). Perona-Garcelán et al. (26) used an event checklist to capture traumatic events in childhood and determined slightly higher prevalence rates of sexual and physical abuse of 46.8 and 49%, respectively. Álvarez et al. (19) ascertained a similar prevalence rate of at least one type of childhood maltreatment of 46.1%. In the study, an event checklist on traumatic events was used and 4 subtypes of childhood maltreatment were defined: physical abuse, sexual abuse, witness to family violence, and psychological abuse, and negligence. In doing so, physical abuse and witness to family violence were assessed using single items and psychological abuse and negligence were requested by an open-ended question. Sexual abuse was assessed using three items, whereby the questions were not explicitly related to abuse by caregivers.

Higher prevalence rates of at least one type of childhood maltreatment can be seen when measures based on broader definitions of childhood maltreatment were used. For example, McCabe et al. (16) used a modified version of the Childhood Adversity Questionnaire [CAQ; (34)] and determined a relatively high prevalence rate of 86.6%. In addition to parental behavior toward the child, the CAQ also covers parental mental health problems and substance use as well as parental separation or loss of a parent and sibling. Further, Schalinski et al. (17) used a more extensive measure, the standardized MACE (35), which, in addition to childhood maltreatment by caregivers, also includes victimization by peers. The authors determined a slightly lower rate of 73% of at least one type of childhood maltreatment, which may be caused by different definitions of childhood maltreatment compared to McCabe et al. (16).

In sum, previous studies often used single items, self-created or modified, and unvalidated measures to capture childhood maltreatment in patients with SSD (e.g., 17, 19, 11), thus the reliability and validity of the data remain unclear. Moreover, some previous studies have focused on sexual and physical abuse (e.g., 24, 25, 26, 29), although emotional forms of child abuse seem to have similarly harmful consequences for mental health as sexual and physical abuse (36). Finally, the conceptualization of measures was often based on different definitions of childhood maltreatment, which complicates the comparability of the data even further (e.g., 22, 17, 11).

In order to facilitate comparability between studies, it is helpful to use standardized, well-established measures. One of the most commonly used standardized measure is the Childhood Trauma Questionnaire (CTQ; 2). The CTQ captures all common types of abusive and neglectful behaviors by family members, such as emotional abuse, sexual abuse, physical abuse, emotional neglect, and physical neglect. Presenting with strong psychometric properties in both clinical and community samples (37, 38), the strength of this instrument is that it allows for a severity rating of the different types of childhood maltreatment (“none to low,” “low to moderate,” “moderate to severe,” and “severe to extreme”) reflecting the fact that types of maltreatment may be continuous phenomena rather than clearly delineated events or experiences (37, 39). However, a dichotomous categorization of the different types of maltreatment using a severity cut-off is indicated to enable the comparison of rates of child maltreatment from studies that applied different instruments based on similar definitions. While the authors do not provide dichotomous thresholds to determine the presence or absence of the different types of childhood maltreatment, previous studies have indicated prevalence rates of childhood maltreatment in SSD patients based on severity ratings. In doing so, Larsson et al. (15) have used the “low to moderate” severity rating as a dichotomous threshold and reported a rate of at least one type of childhood maltreatment of 85%. Other studies have used the more conservative “moderate to severe” threshold and have determined lower prevalence rates between 45 and 73% (25, 28–30). In contrast, some authors have not utilized threshold values based on the original severity ratings, but on empirically determined and validated threshold values as reported by Walker et al. (20, 24, 40). To ascertain these cut-off scores, the CTQ-subscale scores were related to ratings of experts blind for the CTQ-scores who conducted detailed clinical interviews. Experts determined whether participants had a history of clinically significant maltreatment based on the fulfillment of consensus child abuse and neglect criteria (40). These criteria comprised the same definitions of abuse and neglect from which the items of the CTQ subscales were derived. Threshold scores were determined applying receiver operating characteristic (ROC) methods and showed good to excellent sensitivity and specificity (40). Hence, the cut-off scores established by Walker et al. (40) allow for a more accurate and clinically relevant evaluation of the presence of childhood maltreatment while clinical significance of the thresholds according to Bernstein and Fink (41) remains unclear. So far, comprehensive reports of prevalence rates of childhood maltreatment in SSD applying the cut-off scores according to Walker et al. (40) are scarce. While Hassan et al. (20) and Mohammadzadeh et al. (24) determined prevalence rates of the different forms of childhood maltreatment with emotional abuse (45.1 and 54.6%) and physical neglect (52.4 and 56.8%) being most commonly reported, detailed information on co-occurrence of forms of maltreatment were not presented.

With prevalence rates ranging between 45 and 85%, the presented lack of consistency in applying threshold values may be responsible for the wide range of reported maltreatment frequencies while using the CTQ. Therefore, more information on the impact of threshold scores on prevalence rates of childhood maltreatment in SSD is needed to facilitate the comparison of studies using the CTQ.

However, in addition to childhood maltreatment referring to transgressions by caregivers, victims of maltreatment by caregivers are also more likely to experience maltreatment by peers, often referred to as peer victimization (42–45). Peer victimization includes overt victimization (physical aggression or verbal threats), relational victimization (malicious manipulation of relationships, such as social rejection) and reputational victimization (damaging another's peer relationships, for example by spreading rumors) (46). In contrast to the consequences of childhood maltreatment, the effects of peer victimization on mental health have been underestimated until recently, as peer victimization has long been considered a normal, developmental experience. However, a growing body of research indicates that peer victimization is also associated with psychopathology such as depression and social anxiety (46–51). However, only a few studies have investigated the relationship between peer victimization and psychosis, reporting prevalence rates up to 48% in patients with psychotic disorders (17, 52). In addition, peer victimization appears to increase the risk of individual psychotic symptoms (53, 54). Nevertheless, given the paucity of studies that have examined the role of peer victimization in SSD, valid conclusions about the relationship between peer victimization and SSD remain open.

In sum, data on prevalence rates of childhood maltreatment in patients with SSD are rare and very heterogeneous. Only a few studies captured childhood maltreatment systematically and reported prevalence rates of all common types of childhood maltreatment (15, 17, 20, 25, 28, 55, 56). However, different thresholds were used to determine prevalence rates of childhood maltreatment in patients with SSD, resulting in a lack of comparability. Additionally, little is known about peer victimization, as another form of adverse childhood experiences, in patients with SSD. Therefore, we aimed to provide and compare systematically captured data on prevalence rates of all common types of childhood maltreatment in patients with SSD using the standardized and well-established Childhood Trauma Questionnaire (CTQ; 1) and the most frequently used thresholds following the guidance of Bernstein and Fink (41) and Walker et al. (40). We expected to find high prevalence rates of childhood maltreatment in SSD. However, we presumed remarkable differences in prevalence rates depending on cut-off scores established. In particular, we presumed higher prevalence rates for emotional abuse, physical abuse and physical neglect, when rates were calculated using cut-off scores according to Walker et al. (40) due to the fact that the corresponding cut-off scores are less conservative. In addition, we aimed to increase knowledge about peer victimization in SSD by conducting exploratory analyses of the extent of experiences of peer victimization in a sample of SSD patients.

## Materials and Methods

### Participants

48 patients with a primary diagnosis of SSD were recruited in four local outpatient and inpatient psychiatric clinics. In these clinics patients with varying psychiatric disorders were treated due to standard clinical care. Eligible patients were recruited by medical staff members through personal contacts on the wards and during group therapy sessions. Participants were included if they met criteria for any schizophrenia-spectrum-disorder according to the International Classification of Mental and Behavioral Disorders Tenth Version [ICD-10: F20–F29; (57)]. Exclusion criteria were an age below 18 and above 65, an IQ below 85, insufficient knowledge of the German language, acute suicidality, substance-induced psychosis, and psychotic disorder due to (brain-) organic dysfunction. Information on exclusion criteria was obtained from the patients' medical records. Of the 48, 72.9% were inpatients. More than 85% of the sample met criteria for schizophrenia, 2.1% for a brief psychotic disorder, and 12.5% for a schizoaffective disorder. Data collection in inpatients took place after an acute psychotic episode. Prior to assessment, patients were informed about the objectives and the procedure of the study and gave their written informed consent to participate. The study was reviewed and approved by the Ethics Committee of the Bielefeld University and of the Department of Medicine at the Justus Liebig University Giessen, Germany.

### Measures and Instruments

Information about number and type of diagnoses was obtained from the medical records of the clinic where participants received care. Sociodemographic data were collected using a sociodemographic questionnaire. Self-reported data were then validated with data from the medical record (e.g., age, number, and duration of inpatient treatments).

Symptom severity was evaluated using the *Positive and Negative Syndrome Scale* [PANSS; (58)]. The 30-item expert rating assesses symptoms of schizophrenia based on information reported by the patient, direct observations, and observations made by health care staff or family members. The severity of each item for the past 7 days is rated on a scale of 1 (absent), 2 (minimal), 3 (mild), 4 (moderate), 5 (moderate to severe), 6 (severe), to 7 (extreme), resulting in three subscales: positive symptoms (range: 7–49), negative symptoms (range: 7–49) and general psychopathology (range: 16–112). Interviews were carried out by trained research assistants or master level clinical psychologists, whereas the PANSS interviews were conducted only by master level clinical psychologists.

The German version of the self-rating scale *Childhood Trauma Questionnaire* (CTQ; 1) was employed to retrospectively assess the exposure to childhood maltreatment by family members. The 28-item scale captures five types of abusive and neglectful behaviors by family members (emotional abuse, sexual abuse, physical abuse, emotional neglect, and physical neglect). Each item is rated from 1 (never true) to 5 (very often true) with a possible range of subscale scores of 5–25 and a total sum score ranging from 25 to 125.

Peer victimization was retrospectively assessed using the *Fragebogen zu belastenden Sozialerfahrungen* [FBS; (59)] *[Adverse Social Experiences Questionnaire]*. The self-rating scale consists of a list of 22 aversive social situations. Patients can indicate whether they had experienced these situations (rated 1) or not (rated 0) during childhood (age 6–12) or adolescence (age 13–18) resulting in two subscales ranging from 0 to 22 and a total sum score ranging from 0 to 44. The total-score of the FBS presented with a satisfying stability over a 20-month period and construct validity has been confirmed suggesting a good fitness of the instrument [e.g., (49, 50, 59)].

### Statistical Analyses

To allow comparability of different studies, the most common thresholds established by Bernstein and Fink (41) and Walker et al. (40) were applied to determine prevalence rates of childhood maltreatment. While cut-off scores according to Walker et al. (40) allow a dichotomous classification, thresholds reported by Bernstein and Fink (41) can also be used to inform about the severity of childhood maltreatment (“none to low,” “low to moderate,” “moderate to severe,” and “severe to extreme”). The cut-off scores established by Walker et al. (40) were 10 for emotional abuse, 15 for emotional neglect, 8 for physical abuse, 8 for physical neglect, and 8 for sexual abuse. The Bernstein severity thresholds are presented in **Table 3**. Unfortunately, previous studies that have applied severity ratings reported by Bernstein and Fink (41) used different thresholds (“low to moderate” or “moderate to severe”) to determine the frequency of childhood maltreatment. In the present study, the more common and more conservative severity ratings of at least “moderate to severe” were used to obtain a dichotomous classification. To determine the prevalence rates of childhood maltreatment, subjects were divided into groups according to fulfillment of the different types of maltreatment. Furthermore, to provide descriptive data, mean values and standard deviations on childhood maltreatment and peer victimization were calculated. To analyze gender differences in prevalence rates, Pearson's chi-square tests or, for expected cell frequencies below 5, the Fisher's exact test, were performed. *T*-tests for independent samples were used to analyze gender differences in mean scores of all types of childhood maltreatment and peer victimization. All analyses were carried out with SPSS 26.

## Results

The total sample consisted of 48 SSD patients. The average age of the participants was *M* = 39.1 (*SD* = 10.3). Table 1 presents participants' demographics and means on psychopathology.

**Table 1:** Subject characteristics and mean values on psychopathology measures (*N* = 48).

Age, *M (SD*, range)		39.1 (10.3, 20–64)
Gender, % female (*n*)		29.2 (14)
Nationality, % German (*n*)		81.3 (39)
Family status, % single (*n*)		81.3 (39)
Income per month (in euro), *M* (*SD)*, range		811.6 (649.7), 90–2,900
Total duration (weeks) of inpatient treatments, *M* (*SD)*, range		57.8 (61.9), 3–288
Total number of inpatient treatments, *M* (*SD)*, range		8.6 (9.9), 1–46
Positive and Negative Syndrome Scale, *M* (*SD*)		55.2 (14.7)
	Positive Symptoms	13.3 (4.9)
	Negative Symptoms	14.2 (5.9)
	General Psychopathology	27.5 (7.1)
Childhood Trauma Questionnaire, *M* (*SD*)		49.3 (15.5)
	Emotional abuse	11.6 (5.3)
	Emotional neglect	13.6 (5.6)
	Physical abuse	8.1 (4.2)
	Physical neglect	9.2 (3.2)
	Sexual abuse	6.9 (3.7)
Peer victimization, *M* (*SD*)^a^		12.9 (7.7)
	Age 6 – 12	4.9 (4.3)
	Age 13 – 18	8.0 (4.9)

a *Fragebogen zu belastenden Sozialerfahrungen [FBS; (59)] [Adverse Social Experiences Questionnaire, n = 47]*.

Out of the sample of 48 SSD patients, we found 37 participants (77.1%) meeting cut-off scores according to Walker et al. (40) and 28 patients (58.3%) meeting the “moderate to severe” thresholds established by Bernstein and Fink (41) for at least one type of childhood abuse or neglect, as measured by the CTQ subscales (41). The prevalence rates for the different types of childhood maltreatment are presented in Table 2. Using cut-off scores established by Walker et al. (40), 18.8% (*n* = 9) of the participants met threshold level for one subtype, 16.7% (*n* = 8) met threshold levels for two subtypes, 14.6% (*n* = 7) for three subtypes, 16.7% (*n* = 8) for four subtypes, and 10.4% (*n* = 5) for all five subtypes. In addition, 10.4% (*n* = 5) met the threshold level as established by Bernstein and Fink (41) for one subtype, 12.5% (*n* = 6) met threshold levels for two subtypes, 8.3% (*n* = 4) for three subtypes, 18.8% (*n* = 9) for four subtypes, and 8.3% (*n* = 4) for all five subtypes. Table 3 shows the distribution of childhood maltreatment severity for all types of childhood maltreatment assessed with the CTQ (41).

**Table 2:** Prevalence rates of childhood maltreatment based on thresholds as established by Bernstein and Fink (41) and Walker et al. (40) in the present study with SSD patients and in a representative sample of the German population.

	**Present study**, ***N*** **= 48**						**Iffland et al. (60)^**b**^, *N* = 2,500**	**Häuser et al. (61)^**b**^, *N* = 2,504**
	**Walker et al**. **(****40****), % (*****n*****)**			**Bernstein and Fink (****41****), % (*****n*****)**			**Walker et al. (40), % (*n*)**	**Bernstein and Fink (41)^**a**^, % (*n*)**
	**Total**	**Female**	**Male**	**Total**	**Female**	**Male**	**Total**	**Total**
Emotional abuse	47.9 (23)	57.1 (8)	44.1 (15)	43.8 (21)	57.1 (8)	38.2 (13)	10.2 (254)	4.6 (115)
Emotional neglect	37.5 (18)	57.1 (8)	29.4 (10)	37.5 (18)	57.1 (8)	29.4 (10)	13.9 (348)	13.9 (348)
Physical abuse	37.5 (18)	42.9 (6)	35.3 (12)	25.0 (12)	28.6 (4)	23.5 (8)	12.0 (301)	5.6 (139)
Physical neglect	66.7 (32)	71.4 (10)	64.7 (22)	45.8 (22)	50.0 (7)	44.1 (15)	48.4 (1,210)	28.7 (719)
Sexual abuse	25.0 (12)	42.9 (6)	17.6 (6)	25.0 (12)	42.9 (6)	17.6 (6)	6.2 (156)	6.2 (156)
At least one type	77.1 (37)	92.9 (13)	70.6 (24)	58.3 (28)	78.6 (11)	50.0 (17)	33.9 %	–

a *Reported prevalence rates based on the “moderate to severe” thresholds*;

b *Analyses based on the same sample; No significant gender differences were found for CTQ subscales (all p's > 0.05)*.

**Table 3:** Severity thresholds and distribution of childhood maltreatment severity (41) (*N* = 48).

	**Severity rating, % (*****n*****)**															
	**None to low**				**Low to moderate**				**Moderate to severe**				**Severe to extreme**			
	**Thresh-old**	**Total**	**Female**	**Male**	**Thresh-old**	**Total**	**Female**	**Male**	**Thresh-old**	**Total**	**Female**	**Male**	**Thresh-old**	**Total**	**Female**	**Male**
Emotional abuse	≤ 8	45.8 (22)	35.7 (5)	50 (17)	9–12	10.4 (5)	7.1 (1)	11.8 (4)	13–15	14.6 (7)	7.1 (1)	17.6 (6)	≥16	29.2 (14)	50.0 (7)	20.6 (7)
Emotional neglect	≤ 9	18.8 (9)	7.1 (1)	23.5 (8)	10–14	43.8 (21)	35.7 (5)	47.1 (16)	15–17	10.4 (5)	14.3 (2)	8.8 (3)	≥18	27.1 (13)	42.9 (6)	20.6 (7)
Physical abuse	≤ 7	62.5 (30)	57.1 (8)	64.7 (22)	8–9	12.5 (6)	14.3 (2)	11.8 (4)	10–12	8.3 (4)	7.1 (1)	8.8 (3)	≥13	16.7 (8)	21.4 (3)	14.7 (5)
Physical neglect	≤ 7	33.3 (16)	28.6 (4)	35.3 (12)	8–9	20.8 (10)	21.4 (3)	20.6 (7)	10–12	27.1 (13)	28.6 (4)	26.5 (9)	≥13	18.8 (9)	21.4 (3)	17.6 (6)
Sexual abuse	5	58.3 (28)	21.4 (3)	73.5 (25)	6–7	16.7 (8)	35.7 (5)	8.8 (3)	8–12	18.8 (9)	28.6 (4)	14.7 (5)	≥13	6.3 (3)	14.3 (2)	2.9 (1)

As can be seen in Table 1, the mean value of childhood maltreatment in the total sample was 49.3 (*SD* = 15.5). Females had a mean value of 54.57 (*SD* = 16.46) while males showed a mean value of 47.15 (*SD* = 14.86; *t*(46) = 1.53, *p* = 0.13). A significant gender difference was observed for sexual abuse (*t*(46) = 2.00, *p* = 0.05), with higher mean values in females (*M* = 8.57, *SD* = 3.72) compared to males (*M* = 6.29, *SD* = 3.54), while no significant gender differences were found for emotional abuse, emotional neglect, physical abuse, and physical neglect (all *p*'s > 0.05).

Table 1 also shows that the mean value of peer victimization in the total sample was 12.9 (*SD* = 7.7). Female patients had a mean value of 14.1 (*SD* = 10.3) while a mean value of 12.3 (*SD* = 6.5) was obtained for male patients (*t*(45) =0.70, *p* = 0.49). In childhood (age 6–12) a mean value of 4.9 (*SD* = 4.3) was determined in the total sample, female patients showed a mean value of 5.6 (*SD* = 5.5) and male patients a mean value of 4.6 (*SD* = 3.6) (*t*(45) =0.80, *p* = 0.43). In adolescents (age 13–18) a mean value of 8.0 (*SD* = 4.9) was determined in the total sample. A mean value of 8.4 (*SD* = 6.3) was observed for female patients and 7.8 (*SD* = 4.3) for male patients (*t*(45) = 0.41, *p* = 0.69).

## Discussion

With this study we aimed at presenting and comparing prevalence rates of childhood maltreatment in a sample of patients with SSD based on both the original “moderate to severe” severity ratings established by Bernstein and Fink (41) and the empirically determined and validated cut-off scores established by Walker et al. (40). 58.3% of the sample reached threshold for at least one type of childhood maltreatment using “moderate to severe” thresholds established by Bernstein and Fink (41), while 77.1% of the sample reached cut-off score according to Walker et al. (40) for at least one type of maltreatment. Physical neglect (46–67%) and emotional abuse (44–48%) were found to be the most frequently reported types of childhood maltreatment followed by emotional neglect (38%), physical abuse (25–38%) and sexual abuse (25%). In addition, we have run exploratory analyses on peer victimization in SSD and reported descriptive data.

Prevalence rates of childhood maltreatment in SSD show a large variability with our results being in a medium range (Table 4). With respect to the prevalence rates for at least one type of childhood maltreatment in patients with SSD, Mørkved et al. (25) reported a slightly higher rate of 67.3%, while the prevalence rate in the study of Schäfer et al. (28) was higher (73.0%) than our finding (58.3%). Both studies also used the “moderate to severe” thresholds as established by Bernstein and Fink (41) for calculating prevalence rates. A comparison of the prevalence rate with the results of Schäfer et al. (28), however, is limited by the fact that the authors included only female subjects in their study. Generally, females report significantly higher rates of sexual abuse than males (60). This is also reflected in our study in the significantly higher mean score of sexual abuse among females compared to males. Accordingly, when comparing the prevalence rates of the different types of childhood maltreatment in our study with those reported by Schäfer et al. (28), rates of emotional and physical types of childhood maltreatment are similar, while prevalence rates of sexual abuse reported by Schäfer et al. (28) are noticeably higher (37%) than in the present study (25%). This difference may have led to a clearly higher prevalence rate for at least one type of childhood maltreatment reported by Schäfer et al. (28).

**Table 4:** Summary of prevalence rates in SSD patients reported in previous studies (in %).

	**Thresholds**	**CTQ total,** ***M* (*SD*)**	**At least one type**	**Emotional abuse**	**Sexual abuse**	**Physical abuse**	**Emotional neglect**	**Physical neglect**
Present study	Walker et al. (3)	49.3 (15.5)	77.1	47.9	25.0	37.5	37.5	66.7
	Bernstein and Fink (2)		58.3	43.8	25.0	25.0	37.5	45.8
Hassan et al. (20)	Walker et al. (3)	–	–	54.6	34.3	43.2	29.6	56.8
Mohammadzadeh et al. (24)	Walker et al. (3)	–	–	45.1	32.9	40.2	39.0	52.4
Larsson et al. (15)	Bernstein and Fink (2)^a^	–	85.0	63.0	35.0	29.0	67.0	46.0
Li et al. (74)	Bernstein and Fink (2)^a^	42.7 (12.1)	–	31.7	39.9	22.2	58.7	71.7
Haug et al. (75)	Bernstein and Fink (2)	47.2 (18.8)	–	32.0^b^ 26.0^c^	14.0^b^ 30.0^c^	11.0^b^ 15.0^c^	32.0^b^ 26.0^c^	32.0^b^ 33.0^c^
Mørkved et al. (25)	Bernstein and Fink (2)	46.4 (15.5)	67.3	23.1	26.9	17.3	30.8	38.5
Schäfer et al. (28)	Bernstein and Fink (2)	48.5 (18.3)	73.0	40.0	37.0	20.0	43.0	37.0
Shannon et al. (29)	Bernstein and Fink (2)	–	45.0	–	–	–	–	–
Vogel et al. (56)	Bernstein and Fink (2)	45.4 (17.5)	-	46.8	27.8	25.6	62.3	65.8
Wang et al. (76)	Bernstein and Fink (2)	–	71.1^d^ 69.0^e^	10.9^d^ 8.6^e^	27.3^d^ 18.9^e^	19.5^d^ 14.7^e^	25.8^d^ 37.8^e^	63.3^d^ 55.2^e^
Xie et al. (30)	Bernstein and Fink (2)	41.3 (12.4)	47.2	19.4	13.9	11.1	19.4	38.9

*Thresholds according to Bernstein and Fink (41) refers to the “moderate to severe” severity ratings*;

a *The “low to moderate” severity ratings were used as dichotomous thresholds*;

b *Male*;

c *Female*;

d *First episode*;

e *Chronic*.

In contrast to our findings and the studies already mentioned, Shannon et al. (29) reported a smaller prevalence rate for at least one type of childhood maltreatment of 45%, although the authors also used the “moderate to severe” threshold. When considering the findings of Shannon et al. (29) it is important to note that the CTQ, designed to be used as a self-report questionnaire, was used as an interview by Shannon et al. (29). The authors themselves limited the method they used, since maltreatment could have led to general mistrust among those affected. Additionally, mistrust as a common symptom in patients with SSD may have led to an underreported history of maltreatment in a research interview. In contrast, a self-report questionnaire provides anonymity and perhaps an increased sense of confidentiality, which may facilitate disclosure. Taking into account the positive correlation between childhood maltreatment and symptom severity (8, 10, 17) and the assumption that inpatients exhibit higher symptom severity, the place of recruitment as an index for symptom severity, may also be an explanation for the difference. While our results are based on data from a mixed sample of outpatients and inpatients, Shannon et al. (29) only examined outpatients, whereas the higher prevalence rate reported by Schäfer et al. (28) was determined in a sample of inpatients.

Unsurprisingly, in line with previous studies in representative samples (60, 61), the prevalence rates calculated with the cut-off scores established by Walker et al. (40) were higher than the rates calculated using the thresholds established by Bernstein and Fink (41). This is due to the fact that the “moderate to severe” thresholds are as or more conservative than the cut-off scores reported by Walker et al. (40).

Accordingly, as expected, prevalence rates for emotional abuse, physical abuse, and physical neglect were higher when rates were calculated using cut-off scores according to Walker et al. (40) whereas prevalence rates for emotional neglect and sexual abuse were equal to prevalence rates calculated using thresholds as established by Bernstein and Fink (41). In comparison, the “low to moderate” thresholds established by Bernstein and Fink (41) are the least conservative of the thresholds described. In line, applying this thresholds led to elevated prevalence rates of at least one type of childhood maltreatment of up to 85% in a previous study (15).

Comparing our results with those of studies that have used other questionnaires instead of the CTQ, we find that the use of thresholds as reported by Walker et al. (40) has resulted in a prevalence rate, which is similar to those of other studies with similarly broad definitions of childhood maltreatment (e.g., 19, 20). The comparatively lower prevalence rate we determined based on the thresholds according to Bernstein and Fink (41) can be classified between those of previous studies. It is lower than those of previous studies with similarly broad definitions of childhood maltreatment (e.g., 19, 20) but, as expected, it is higher than rates in previous studies that have limited the investigation to sexual or physical abuse (e.g., 25, 26, 29). Thus, comparing studies that used different instruments remains difficult when different definitions of childhood maltreatment were used.

Considering the prevalence rates of the various forms of maltreatment, emotional abuse and physical neglect were most frequently reported in our study. These results are similar to those of previous studies that have used the CTQ to capture childhood maltreatment in SSD (15, 20, 24, 28, 55, 56, 62). Physical neglect and emotional types of childhood maltreatment were also most commonly reported in a representative sample of the German population (60, 61). However, prevalence rates of all types of childhood maltreatment in our SSD sample were clearly higher and more than twice as high for at least one type of childhood maltreatment compared to the prevalence rates in the representative sample (see Table 2). This highlights the relevance of childhood maltreatment in SSD.

However, a validation study from the general German population indicated that the subscale for physical neglect shows high intercorrelations with the other subscales and a weak internal consistency (63), thus elevating the risk of an over-estimation of prevalence rates for physical neglect. When following the authors' recommendation to apply the subscale “physical neglect” with caution or even to exclude it from analyses, the present results indicate the specific significance of emotional forms of maltreatment in patients with SSD. Because similar distributions were also found in studies examining other psychiatric disorders such as affective or anxiety disorders (49, 50, 64), it may be suggested that the distribution and significance of the different types of childhood maltreatment are not limited only to patients with SSD.

Although exploratory, the present study was among the first to examine associations of peer victimization and SSD. Levels of peer victimization presented in our SSD sample (*M* = 12.9, *SD* = 7.7) were consistent with previous studies that used the FBS to capture peer victimization in different clinical samples [e.g., (65, 66)]. Most notably, Iffland et al. (67) as well as Sansen et al. (50) showed that levels of peer victimization in a healthy sample [*M* = 7.7 (*SD* = 4.8) and *M* = 9.0 (*SD* = 6.6)] were substantially lower than in patients with varying psychiatric disorders [*M* = 16.5 (*SD* = 8.7) and *M* = 12.7 (*SD* = 8.5)]. Due to a lack of cut-off scores for the FBS, no actual prevalence rate of peer victimization could be reported in this study, resulting in a lack of comparability with other studies. For instance, Schalinski et al. (17) used the German version of the MACE Scale (68) to assess childhood maltreatment and peer victimization in SSD. The authors reported prevalence rates of peer victimization up to 48% in patients with SSD suggesting that experiences of peer victimization are as common as childhood maltreatment in patients with SSD.

### Strengths and Limitations

To our knowledge, the present study is the first to present prevalence rates for childhood maltreatment in SSD based on both cut-off scores according to Walker et al. (40) and Bernstein and Fink (41), which allows a thorough evaluation of data for childhood maltreatment in SSD in future research. Accordingly, our results provide information on how conservative the investigated cut-off scores are and may thus facilitate the decision on which cut-off scores to use in future research. Additionally, explorative data of peer victimization were presented in this study. With respect to the alarming prevalence rates, the current study has practical implications. Prevention of childhood maltreatment is highly relevant - with respect to our explorative analyses maltreatment here refers to victimization by caregivers and by peers. In particular, sensitizing, informing, and training relevant (e.g., caregivers, educators, teachers) could help identifying children at risk for childhood maltreatment, especially emotional types of childhood maltreatment, and latter impacts on mental health. Moreover, the routine collection of any form of childhood maltreatment history from all users of mental health services is recommended. In this regard, when using standardized self-reports such as the CTQ, more sensitive cut-off scores, such as those established by Walker et al. (40), should be used in order to minimize false-negative results. Finally, patients who have been abused or neglected by caregivers during childhood or adolescence should receive the offer of appropriate psychosocial treatments. At this point, the present study emphasizes that this also applies to patients with more genetically-conceptualized disorders such as SSD.

However, the present study has several limitations. First of all, memory errors and biases may have influenced the results of our study. Particularly, the retrospective nature of the CTQ and FBS may have led to inaccuracy of reports. Memory biases may be a particular problem in SSD, since patients could have difficulties in reality testing. This may be even more true in not fully clinically stabilized SSD patients as tested in our study. However, Read et al. (69) suggested that retrospective self-reports can be considered reliable even if participants with a history of psychosis were included. Fisher et al. (70) also found that reports of childhood maltreatment were not associated with current severity of psychotic symptoms. However, co-morbid disorders or sociodemographic characteristics may have influenced the prevalence rates calculated in our study. Accordingly, Bonoldi et al. (71) indicated that childhood maltreatment is moderated by substance abuse and age. Further, it is uncertain whether our sample of patients with SSD is representative of all patients with SSD, especially since our sample size is small and includes relatively few females, as it is often the case in SSD research. Therefore, subgroup analyses, for example exploration of gender differences, were limited in our study and should be investigated in future research. In addition to the small sample size, the present study lacks a healthy control group. Hence, conclusions regarding increased prevalence rates in SSD patients and its role in the etiology of the disorder have to be drawn with caution. Finally, although the German version of the CTQ seems to be a reliable and valid measure for childhood maltreatment in clinical samples (72, 73), a validation study from the general German population has indicated that the subscale for physical neglect shows high intercorrelations with the other subscales and a weak internal consistency (63), therefore the authors recommend applying the physical neglect subscale with caution.

## Conclusion

Childhood maltreatment is a common phenomenon in SSD, even though methodological details, especially cut-off scores, have a substantial impact on the prevalence rates that are determined. Therefore, the methodology of studies should be closely examined when drawing conclusions from presented prevalence rates.

## Data Availability Statement

The data sets presented in this article are not readily available. To preserve the anonymity of participants, only aggregated data can be provided. Requests for access to datasets should be directed to Angelina Weitkämper, angelina.weitkaemper@uni-bielefeld.de.

## Ethics Statement

The studies involving human participants were reviewed and approved by Ethics Committee of the Bielefeld University and the Ethics Committee of the Department of Medicine at the Justus Liebig University Giessen. The patients/participants provided their written informed consent to participate in this study.

## Author Contributions

AW made substantial contributions to the conception and design of the work, the acquisition, analysis, and interpretation of the data, drafted, revised, and approved the manuscript, and ensures the accuracy and integrity of any part of the work. MK, JI, MD, HK, and FN contributed to the conception of the study and revised the manuscript critically for important intellectual content. MK, JI, MD, and HK made substantial contributions to data acquisition. BI was the chief investigator for this study, contributed to the conception of the study, supervised data analyses, participated in the interpretation of the data, and critically revised the manuscript for important intellectual content. All authors contributed to the article and approved the submitted version.

## Conflict of Interest

The authors declare that the research was conducted in the absence of any commercial or financial relationships that could be construed as a potential conflict of interest.
